# Body adiposity index performance in estimating body fat in a sample of severely obese Brazilian patients

**DOI:** 10.1186/s12937-015-0119-8

**Published:** 2015-12-30

**Authors:** Giliane Belarmino, Lilian Mika Horie, Priscila Campos Sala, Raquel S. Torrinhas, Steven B. Heymsfield, Dan L. Waitzberg

**Affiliations:** 1 Department of Gastroenterology, Surgical Division, LIM 35.University of São Paulo School of Medicine, São Paulo, Brazil; 2 Pennington Biomedical Research Center, Baton Rouge, Louisiana USA; 3 Nutrition Laboratory and Metabolic Surgery of the Digestive Tract, LIM 35 University of São Paulo, Medical School. Dr. Arnaldo avenue, 455, Cerqueira César. Postal code: 01246-903 São Paulo, Brazil

**Keywords:** Obesity, Body fat, Body composition, Nutritional assessment, Body adiposity index, Air displacement plethysmography

## Abstract

**Background/objectives:**

The body adiposity index (BAI) estimates the amount of body fat (BF) in humans. In Mexican-American and African-American populations, BAI has performed better than body mass index (BMI). The aim of this study was to evaluate the performance of BAI in estimating percentage (BF%) in severely obese Brazilian patients, with air displacement plethysmography (ADP) used as the reference method.

**Subjects/methods:**

Estimation of BF% by ADP, anthropometric measurements (height, abdominal and hip circumferences, body weight, and BMI) and BAI calculation were performed in 72 obese subjects (BMI ≥ 30 kg/m^2^) aged 30–55 years.

**Results:**

The mean BF% estimates ± standard deviation were 52.1 ± 5.7 % for ADP and 47.7 ± 7.4 % for BAI, with a positive Pearson correlation (*r*_p_ = 0.66) and a positive Lin’s concordance correlation (*r*_c_ = 0.479) observed between these methods. The 95 % limits of individual agreement between BAI and ADP ranged from -5.769 % to 16.036 %, with BAI exhibiting an average positive bias of 5.13 % compared to the reference method. For each studied variable, BAI exhibited a systematic bias, as evidenced by a tendency for low BF% values to be overestimated.

**Conclusion:**

For Brazilian patients with severe obesity, BAI does not provide an accurate estimate of BF%.

## Introduction

Obesity is a multifactorial disease that has reached epidemic levels worldwide [1, 2]. Obesity is characterized mainly by excessive body fat (BF) that is related to the development of major comorbidities, such as type 2 diabetes mellitus. BF may be influenced by demographic variables, such as age, gender, and ethnicity. Universally, body mass index (BMI) has been used to classify obesity as mild (grade I), moderate (grade II), and severe (grade III), with respective values of 30-34.9, 35-39.9 and ≥ 40 kg/m^2^ [2, 3]. However, BMI may not fully reflect the amount and distribution of BF, and may not clearly distinguish the fat-free mass (FFM) compartment. Gallagher et al. [4] previously showed that BMI is strongly influenced by age and gender, and population studies that use BMI as an indicator of BF must be interpreted in light of this finding.

In obese subjects, BF assessment gains precision by using more specific methods for body composition evaluation. In particular, air displacement plethysmography (ADP) has been validated as a reference method in estimating fat mass in severely obese patients [5]. ADP is an accurate, noninvasive, rapid, and reliable tool for assessing BF, although its routine use in clinical practice is limited mainly due to its high cost [6, 7]. Recently, the body adiposity index (BAI) was developed as an alternative to BMI and other available tools for body composition assessment and it was found to be more sensitive in estimating BF% than BMI, compared to the reference dual-energy X-ray absorptiometry (DXA) method [8].

BAI, as an estimate of body fat percentage (BF%), is calculated using a simple equation that includes only hip circumference and body height. The rationale for this approach derives from a study by Bergman et al. [8] where a significant Pearson correlation coefficient (rp) was observed between each of these two variables and DXA-determined BF% (rp = 0.60 and -0.52, respectively). In this study, BAI was used to estimate the BF% of Mexican-American adult, and it was successfully validated in adult African-American men and women. Since BAI exhibited good performance during its validation in a population that differed from the one with which it was developed, Bergman et al. suggested that the BAI would not need further adjustments for characteristics such as gender and age to improve its performance. Thus, BAI was purported to be a potentially rapid, inexpensive, and noninvasive assessment tool for use in clinical practice.

However, other studies have shown that the performance of BAI has not been consistent in other populations with characteristics different from those used for its development and validation. For example, in adolescents aged 12–16 years, Thivel et al. [9] found a weak association between the BF% estimates determined by BAI versus DXA. In collegiate women, Esco et al. [10] cross-validated BAI with DXA as the reference method, and the former was found to be associated with large individual errors when predicting BF% in female athletes. BAI also exhibited a tendency to overestimate BF% at lower levels. Consequently, the investigators concluded that BAI should not be used for estimating BF% in athletic women. Furthermore, in a study of overweight and obese postmenopausal Caucasian women, BAI underestimated BF% by up to 7.56 % compared with DXA [11].

It is also possible that BAI results may be influenced by the amount of BF% present. For example, Chang et al. [12] observed that the BAI method had a tendency to overestimate and underestimate BF% in men and women, aged 55–96 years, that presented with BF% estimated by DXA at < 15 % and ≥ 40 %, respectively. In light of these findings, it is striking that both of the databases that were used for the development and validation of the BAI included groups of subjects with varying degrees of BF% (BMIs average: ~ 30 kg/m^2^, ranging from normal to obese) [8]. It is worth noting that more severely obese patients are at higher complications risk associated with excess body fat. Their large size and weight limits de performance of simple (such as skin fold) and more sophisticated (such as DXA) methods for the clinical BF% estimation. Therefore, we tested whether BAI could have a good performance to estimate BF% in severely obese Brazilian patients.

## Subjects and methods

### Patients

For this study, 72 adult severely obese Brazilian patients (53 female, 19 male), who were candidates for bariatric surgery and each of whom had a BMI ≥ 30 kg/m^2^, were recruited from the Digestive Tract Surgery Service at the Hospital das Clínicas - University of São Paulo School of Medicine, São Paulo, Brazil. The exclusion criteria were: neurologic or psychiatric conditions; substance abuse; lactating or pregnant women; HIV-positive or cancer patients; clinically detectable edema; physical amputations; chronic and acute diseases of the liver, lung, kidney, and heart; and refusal to give informed consent. All study procedures were performed according to the ethical standards of the World Medical Association’s Declaration of Helsinki, approved by the institutional ethics review board (1069/05 and 1011/09) and this study is part of a major trial assessing body composition in obese individuals before and after bariatric surgery, registered at (NCT01251016). Written informed consent was obtained from each patient prior to trial participation.

The sample size calculation was based on a study by Geliebter et al. [13], which estimated the Pearson correlation coefficient between BAI and ADP at 0.73. With 90 % power and 5 % significance level, it was estimated that a sample of 62 subjects were sufficient to test the null hypothesis against a *r*_p_ of 0.5. The sample size was calculated in the G*Power software package (version 3.1.9.2, Heinrich Heine University, Dusseldorf, Germany According to the Brazilian Institute of Geography and Statistics (IBGE), sample ethnicity was self-reported as race by considering skin color for classification into the following categories: white, black/brown, yellow and indigenous [14].

### Measurements

Body weight (kg, minimal variation of 10 g) was measured by using the weekly-calibrated body weight scale of the ADP system (Bod Pod body composition system Life Measurement Instruments, Concord, CA, USA), with the patient standing in the center of the scale platform, barefoot, and wearing only underwear. Body height (cm) was obtained with a stadiometer (Sanny, São Paulo, Brazil), with the patient standing, barefoot with the heels together, back upright, and arms stretched next to the body. Hip circumference (cm) was measured by positioning a measuring tape in the horizontal plane at the greatest circumference of the buttocks [8, 15]. Waist circumference was measured at the trunk midway between the lower costal margin (bottom of the lowest rib) and the iliac crest (top of the pelvic bone) with the subject standing with his/her feet 25–30 cm apart. The measurement was taken by fitting the tape snugly, without compressing the underlying soft tissue. Circumference was measured to the nearest 0.5 cm; at the end of a normal expiration [16]. The following equation was used to estimate BF: BF% ≈ BAI = [(hip circumference (cm)/height (m)^1.5^) − 18] × 100. In addition, BMI was calculated as body weight (kg) / height (m)^2^ and classified according to the World Health Organization scoring system [2, 3].

### ADP

Using the Bod Pod, ADP was performed to estimate total BF (kg). In the ADP method, the inverse relationship between pressure and volume proposed by Boyle (P1 × V1 = P2 × V2) is used to determine the body volume (BV). BV is used to calculate density (D = M/V), and BF% is then calculated using Siri’s equation: BF% = (4.95/D – 4.5) × 100, where D = density. All measurements and calculations are automatically performed by the system software, and they are based on air volume and pressure variations inside the Bod Pod chamber when occupied and not occupied by the patient [17, 18]. During ADP evaluations, the patients wore only underwear and a cap to keep their hair fastened, and they remained in a sitting position inside the chamber [18]. Metallic objects, such as earrings, rings, chains, and body piercings were not allowed.

### Statistical methods

BF% estimates are expressed as the mean ± standard deviation (SD). Other continuous variables are expressed in terms of statistical position (minimum, maximum, median, or mean) and scale (SD or interquartile range). Gender, race, and categorical variables are expressed in absolute and relative frequencies. Relationships between variables were analyzed by scatter plots, and Pearson correlation coefficients were calculated [19]. Lin’s concordance correlation coefficient (*r*_c_) was used to assess the reproducibility between BAI and ADP. The 95 % limits of agreement between the BAI and ADP and BMI and ADP were determined using the Bland-Altman method [20]. Statistical analyses were performed in the R software package (version 3.1.0, R Development Core Team, 2014). For construction of graphs, the ggplot2 package was used [21]. A *p* value < 0.05 was considered statistically significant.

## Results

**Fig. 1:**
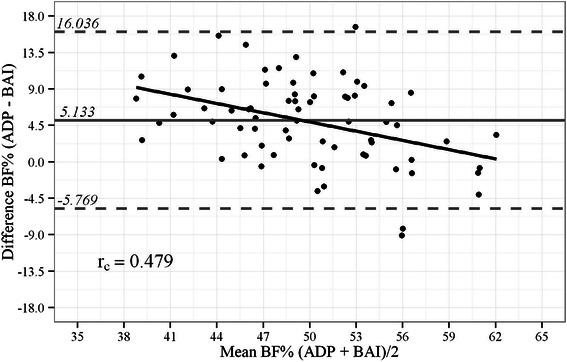
Bland-Altman plot showing limits of agreement between BF% by BAI vs. ADP. Bold continuous line indicates observed average agreement. Continuous line indicates line of perfect average agreement. Dashed lines indicate 95 % limits of agreement. Lin’s concordance correlation coefficient (*r*_c_) is shown

**Fig. 2:**
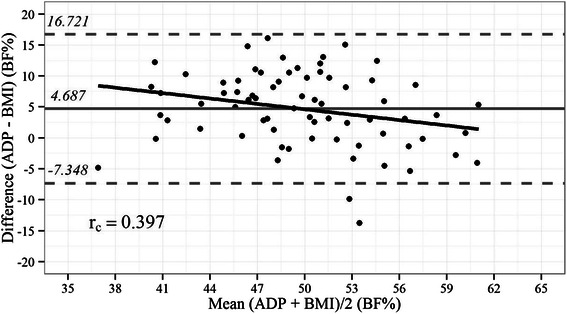
Bland-Altman plot showing limits of agreement between BF% by BMI vs. ADP. Bold continuous line indicates observed average agreement. Continuous line indicates line of perfect average agreement. Dashed lines indicate 95 % limits of agreement. Lin’s concordance correlation coefficient (r_c_) is shown

Table 1 provides the baseline demographic and anthropometric data of the 72 obese patients assessed. The mean BF% estimates measured by ADP and BAI were 52.05 ± 5.66 % and 47.65 ± 7.38 %, respectively, with *r*_p_ = 0.66 and *r*_c_ = 0.479. The 95 % limits of individual agreement between BAI and ADP were −5.77 % to 16.04 % (range: 21.8 %), as shown in Fig. 1. These limits of individual agreement were higher than those found between BMI and ADP (−7.34 % to 16.72 %, range: 24.1 %; r_p_ = 0.39), as shown in Fig. 2. However, the BAI exhibited an average positive bias of 5.13 % compared to the reference method.

**Table 1:** Demographic and anthropometric data of obese patient sample

	Gender						
	Female (*n* = 53)		Male (*n* = 19)		Total (*n* = 72)		*p* value
	n	*%*	n	*%*	N	*%*	
Age (years)	44,7	11,8	36,7	12,2	42,6	12,3	0,015 ^a^
Body weight (kg)	118,2	20,7	154,08	27,37	127,68	27,5	<0,001 ^a^
Height (m)	1,6	0,1	1,8	0,1	1,6	0,1	<0,001 ^a^
BMI (kg/m^2^)	46,8	6,4	49,4	7,4	47,4	6,7	0,146 ^a^
Abdominal circumference (cm)	132,1	14,6	152,3	16,1	137,5	17,4	<0,001 ^a^
Hip circumference (cm)	134,3	13,8	137,6	13,9	135,2	13,8	0,382 ^a^
Waist/hip ratio	1	0,1	1,1	0,1	1	0,1	<0,001^a^
Race^a^ white	34.0	64,2	12	63,1	46	63,9	0,644 ^b^
Race^a^ black/brown	19.0	35,8	7	36,9	26	36,1	
BF% – ADP	53,4	4,7	48,6	5,9	52,1	5,4	0,001^a^
BF% - BAI	49,2	6,5	40,8	5,4	47	7,2	<0,001 ^a^

^a^Student *t*-test. ^b^Chi square. *BMI* body mass index, *ADP* air displacement plethysmography, *BF%* body fat percentage, *BAI* body adiposity index

**Fig. 3:**
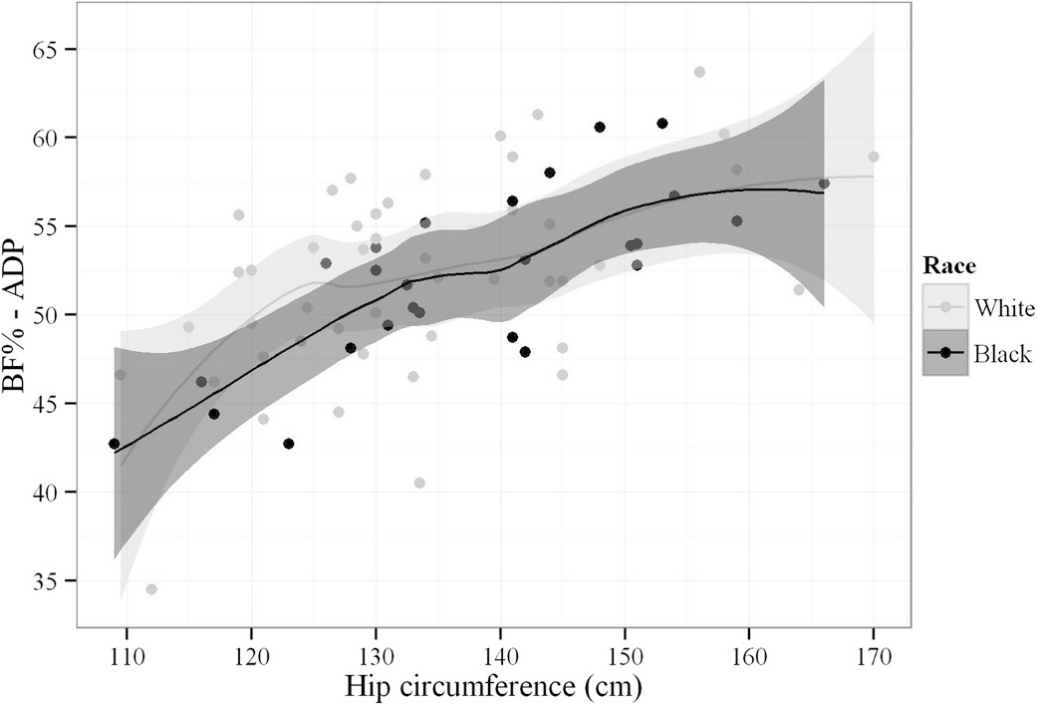
Scatter plot of hip circumference vs. BF% by ADP among white and black/brown obese Brazilian patients. Solid line represents the Local Polynomial Regression fitting that is bounded by the 95 % confidence band

The correlation coefficients between the independent variables and the BF% estimates by ADP, as well as their respective 95 % confidence intervals, are listed in Table 2. Except for age and height, all evaluated variables showed significant correlations with the BF% estimates by ADP, although these coefficients were low with reasonable values (> 0.5) only for BMI, hip circumference, and BAI (*r*_p_ = 0.529, 0.59, and 0.664, respectively). In addition, no significant correlations between the ADP-determined BF% estimates and these variables were observed with respect to race, even for hip circumference, which was presently correlated to ADP and also correlated to DXA during the BAI development (Fig. 3).

**Table 2:** Pearson correlation coefficients between ADP-estimated BF% and independent variables

Variable	*r*_p_	Confidence interval (95 %)		*p* value
		Lower	Higher	
Age (years)	0.137	-0.098	0.357	0.252
Body weight (kg)	0.259	0.03	0.463	0.028
Height (m)	-0.156	-0.374	0.078	0.19
BMI (kg/m^2^)	0.529	0.338	0.677	< 0.001
Abdominal circumference (cm)	0.27	0.041	0.472	0.022
Hip circumference (cm)	0.59	0.414	0.723	< 0.001
Waist/hip ratio	-0.24	-0.447	-0.009	0.042
BF%–BAI	0.664	0.511	0.776	< 0.001

Abbreviations: *ADP* air displacement plethysmography, *BF%* body fat percentage, *BMI* body mass index, *BAI* body adiposity index, *r*_*p*_ Pearson correlation coefficient

## Discussion

For estimating BF% in severely obese Brazilian patients, BAI was found to provide differing values from those estimated by ADP, especially for patients with lower BF% values. Moreover, in the latter case, BAI exhibited a positive systematic bias. The correlation between BF% values estimated by ADP and BAI was significant, probably because similar variation patterns exist for both methods. However, in the Bland-Altman plots that were generated, the limits of agreement between the two assessment tools were large and BAI exhibited a positive bias compared to the reference method. These findings suggest that, in the population studied, the estimates of BF% by BAI included a substantial degree of error that could potentially lead to an overestimation of BF%.

In previous studies, BAI appeared to be more accurate when measuring individuals with a fat mass that ranged from 20 % to 30 % of their total body weight. However, in subjects with total fat amounts above or below this range, BAI exhibited the potential to underestimate or overestimate BF% [12]. In the present study, the Brazilian patients that were evaluated had BF% values (as determined by ADP) that were greater than 30 % of their total body weight, and BAI exhibited a tendency to overestimate BF%. In addition, the lack of a significant correlation between hip circumference and ADP-determined BF% with respect to race may be due to the ethnic background diversity of the Brazilian population which includes a mixture of Amerindian, European, and African genetic backgrounds and implies in a less accurate determination of ethnicity by race based on skin color [22].

Previously, in a study [23] of Brazilian women (mean BF% = 36.9 ± 6.2 %) where DXA was used as the reference method, BAI demonstrated poor concordance and low accuracy and precision. Furthermore, the mean BAI-determined BF% was statistically lower than that measured with DXA. Similarly, application of the mean BAI-determined BF% in the present study resulted in underestimates of BF%. In a study by Geliebter et al. [13], the limits of agreement between BAI and the reference method (either DXA or ADP) were wider than those between BMI and the reference method. Furthermore, the range of the confidence limits between BAI and ADP in their study (−18.47 % to 8.49 %, range: 27.0 %) are similar to the confidence limits in the present study. The authors concluded that although BAI appears to be a reasonable index of BF%, it is not an appropriate substitute for BMI in evaluating severely obese women. Although in our severely obese Brazilian patients the BAI performance was discreetly better than the BMI performance to estimate BF%, our findings support the conclusion of Geliebter et al. by not displaying similar results of this measuring between BAI and ADP. When BAI performance has been studied in other clinical settings of obesity, such as those involving Down syndrome and chronic renal disease, poor performance of BAI in comparison to DXA was observed [24]. In adult Down syndrome patients, BF% by DXA was significantly lower than that estimated by BAI, with BAI overestimating the BF% by 2.65 %. In contrast, BAI estimated BF% with high accuracy in nondialyzed adult chronic kidney disease patients compared to DXA [25]. It should be noted that the BMIs in the latter group of patients (25.0–29.9 kg/m^2^) were lower than those in the present study population.

Here, similarly to Bergman et al. study a significant, but relatively low correlation between BF% determined by the reference method and hip circumference was observed. In addition, an inverse correlation between BF% determined by the reference method and height was also presently found but it was not significant, differently of Bergman et al. findings. The lack of a significant inverse correlation between ADP-estimated BF% and height observed by us may be because we included only severe obese patients, while Bergman et al. included subjects with BMI ranging from normal to obese values during the BAI development and validation. Taller subjects are likely to have a higher area for adiposity distribution than those shorter, but in severe obese patients the excessive fat amount and its general main concentration in abdominal area may neutralize this effect. Consequently, other variables (such as abdominal circumference) seem more relevant than height to estimate BF% in this population.

In addition, it is possible that height could be responsible for the neutral effect of gender on BAI performance, which accounted to a lack of concern to include a similar number of men and women in our study. Men are generally taller, with a consequent higher area for adiposity distribution than women. Therefore, by considering height for calculation, BAI may neutralize this difference between genders. The lack of an effect of height on BF% estimation observed by us suggests that a new formula adjusted by gender may be required for BF% estimation in severely obese subjects. Furthermore, is possible that the inclusion of other anthropometric measurements into the BAI equation may provide correction factors to obtain more accurate BF% estimates among obese Brazilians. The development of a new BAI equation to estimate BF% in the Brazilian population is of clinical interest, given the reported advantages of the BAI method.

A limitation of the present study was the relatively small sample of Brazilian obese patients with a BMI of ≥ 30 kg/m ^2^ (range: 30–40 kg/m^2^). In addition, the inclusion of severe obese patients in our sample implied in using ADP, and not DXA, as the gold standard method for BF% estimation. While ADP has been shown to be suitable to adequately access body composition in patients with BMI over 40 kg/m^2^, the use of DXA usually is applied only in patients with limited weight and size [26, 27]. In an obese population similar to our study, Hames et al. (2014) found a strong agreement between DXA and ADP performance in estimating fat content regardless of its expression unit (kg and %) [28].

## Conclusion

BAI was shown to be a simple method for calculating BF%, and was also noninvasive and relatively inexpensive. However, the performance of BAI was found to be strongly influenced by the characteristics of our patient population. Specifically, BAI exhibited poor performance in obese patients with a BMI greater than 30. While the present findings in no way detract from the potential for the clinical application of BAI, they do suggest that BAI may not be adequate for estimating BF% in severely obese Brazilian patients.
